# Efficacy and Safety of Aldosterone Synthase Inhibitors for Resistant Hypertension: A Systematic Review and Meta-Analysis

**DOI:** 10.31083/RCM39555

**Published:** 2025-08-19

**Authors:** Ying Zhang, Chuanying Huang, Lidi Liu, Miaomiao Wu, Haiqi Song, Shize Wan, Yonglang Cheng, Xiaoyang Liao, Dongze Li

**Affiliations:** ^1^General Practice Ward/International Medical Center Ward, General Practice Medical Center, West China Hospital, Sichuan University, 610041 Chengdu, Sichuan, China; ^2^Department of Emergency Medicine, General Practice Medical Center, National Clinical Research Center for Geriatrics, West China Hospital, 610041 Chengdu, Sichuan, China; ^3^King's University College at Western University, London, ON N6A 2M3, Canada; ^4^Teaching & Research Section, General Practice Medical Center, West China Hospital, Sichuan University, 610041 Chengdu, Sichuan, China

**Keywords:** aldosterone synthase, inhibitor, resistant hypertension, risk

## Abstract

**Background:**

Compared to patients with controllable hypertension, those with resistant hypertension (RH) have a higher incidence of cardiovascular complications, including stroke, left ventricular hypertrophy, and congestive heart failure. Therefore, an urgent need exists for improved management and control, along with more effective medications. Aldosterone synthase inhibitors (ASIs) are newly emerging drugs that have gradually attracted an increasing amount of attention.

**Methods:**

The Cochrane Library, PubMed, Embase, and ClinicalTrials.gov databases were systematically searched to identify all literature on ASIs and resistant hypertension. Additionally, the reference lists of the included articles were manually searched. The quality of the identified studies was assessed using the Cochrane Bias Risk Tool.

**Results:**

This study comprised four randomized controlled trials (RCTs), involving 776 participants. Different doses of ASIs were used, with treatment durations ranging from 7 to 12 weeks. The selected study population included individuals with resistant hypertension and healthy adults. Systolic blood pressure (SBP) had a pooled effect size of standardized mean difference (SMD) = –0.24, with a 95% confidence interval (CI) of [–0.46, –0.03], indicating a statistically significant difference (*p* = 0.026); however, diastolic blood pressure (DBP) had a pooled effect size of SMD = –0.13, with a 95% CI of [–0.40, 0.15], indicating no significant difference (*p* = 0.359). Similarly, subgroup analyses yielded comparable results. Notably, the risk of adverse events in the ASI group was greater than that in the control group, with a risk ratio of 1.32 and a 95% CI of [1.04, 1.66], indicating a significant difference (*p* = 0.02). There was no statistically significant difference in severe adverse events between the treatment group and the control group (*p* = 0.532).

**Conclusions:**

ASIs have shown benefits in controlling SBP in patients with resistant hypertension, although their effects on DBP appear to be limited. Given the observation period of only 12 weeks, the potential for increased adverse event risks with their use warrants further attention. Considering the relatively small number of trials included and the limited sample size in this study, future research should focus on expanding the sample size and extending the follow-up duration to more precisely define the clinical role and value of ASIs. Additionally, further investigation into the underlying mechanisms of action of these inhibitors is necessary to provide theoretical support for optimizing treatment strategies for resistant hypertension and related conditions.

## 1. Introduction

Patients with resistant hypertension (RH) are individuals who have blood pressure levels above the target range even after being prescribed maximal tolerated doses of three or more different classes of antihypertensive medications, including long-acting calcium channel blockers, angiotensin II receptor blockers, angiotensin-converting enzyme inhibitors, and diuretics [1, 2, 3]. In adults with hypertension, RH is considered a relatively common condition, but estimating its true prevalence is challenging due to the lack of data [4, 5], particularly regarding the exclusion of white coat effects and medication nonadherence. Data from cross-sectional and hypertension outcome studies suggest that the estimated prevalence of RH in the general hypertensive population ranges from 10% to 20% [6]. Risk factors for RH include Black ethnicity, older age, male sex, obesity, the presence of diabetes, and chronic kidney disease [7]. Compared to patients with controlled hypertension, those with RH have a higher incidence of cardiovascular complications, including stroke, left ventricular hypertrophy, and congestive heart failure, by approximately 50% [8].

The pathophysiological mechanism of RH is related to fluid retention and the aldosterone excess state [9]. Excessive sodium and fluid retention, activation of the renin-angiotensin-aldosterone system, and enhanced sympathetic nervous activity appear to play crucial roles in the development of treatment RH. Emerging evidence also highlights the contributions of arterial stiffness and potential gut dysbiosis to its pathogenesis [10]. In the past, when common medications were ineffective, the addition of mineralocorticoid receptor antagonists (MRAs), such as spironolactone or eplerenone, was typically considered [11]. However, the widespread use of spironolactone is limited due to its side effects, especially those related to off-target steroid receptor-mediated effects and hyperkalemia [12]. Recently, several novel compounds targeting relevant pathological pathways have emerged, including the dual endothelin receptor antagonist apresoline, the aldosterone synthase inhibitor (ASI), and the nonsteroidal mineralocorticoid receptor antagonist finerenone [13]. Inhibiting aldosterone synthesis may become a new option for RH treatment, and the benefits of inhibiting aldosterone synthesis may not be limited to resistant hypertension alone, as elevated aldosterone levels are associated with the pathobiology of pulmonary arterial hypertension, obesity, insulin resistance, and metabolic syndrome [14]. Recently, several highly selective ASIs, such as baxdrostat, lorundrostat, and dexfadrostat, have emerged. These inhibitors are capable of lowering aldosterone levels without significantly altering cortisol levels [15].

In summary, this study aimed to comprehensively retrieve all research on the use of ASIs to treat RH and endeavored to analyze the efficacy and safety of ASIs in treating RH. The objective of this study was to provide scientific evidence for future new drug selection for RH patients.

## 2. Materials and Methods

This meta-analysis was conducted in accordance with the Preferred Reporting Items for Systematic Reviews and Meta-Analyses (PRISMA) guidelines. The PubMed, Embase, ClinicalTrials.gov, and Cochrane Library databases were searched from inception to February 15, 2024, to identify randomized controlled trials. There were no language restrictions. This study has been registered in the International Platform of Registered Systematic Review and Meta-analysis Protocols (INPLASY) with registration number INPLASY202430063.

### 2.1 Literature Search

The Cochrane Library, PubMed, Embase, and ClinicalTrials.gov databases were searched. Additionally, the reference lists of the included articles were manually screened. The following keywords were used in the search: “Aldosterone synthase”, “Inhibitor”, “Resistant hypertension”, and “randomized controlled trial”.

### 2.2 Inclusion and Exclusion Criteria

The inclusion criteria were as follows: (1) randomized controlled trials (RCTs) or clinical trials; (2) intervention with ASIs; (3) hypertension indicators such as systolic blood pressure, diastolic blood pressure, and safety indicators of drugs; and (4) studies involving adults. Effect sizes were calculated using existing data. The exclusion criteria were as follows: animal studies, review articles, and conference abstracts.

### 2.3 Data Extraction

Two lead authors independently screened the titles and abstracts of the retrieved studies and reviewed the full texts to determine whether the studies met the inclusion criteria. Any discrepancies were resolved by the corresponding author. The following data were extracted into electronic spreadsheets: (1) first author’s name, (2) publication year, (3) country where the study was conducted, (4) study design, (5) number of participants in the intervention and control groups, (6) intervention protocol, (7) drug dosage, (8) duration of intervention, (9) age and gender of study participants, as well as systolic blood pressure, diastolic blood pressure, adverse events, etc.

### 2.4 Quality Assessment

This meta-analysis utilized Cochrane standards to assess the quality of the studies involved to determine the levels of selection, performance, detection, attrition, and reporting biases for each trial. Each domain was assessed to determine the level of bias within the defined area. The quality of the studies was evaluated using the Cochrane Risk of Bias Tool for RCTs [16]. Six dimensions were assessed, including random sequence generation (selection bias), allocation concealment (selection bias), blinding of outcome assessment (detection bias), incomplete outcome data (attrition bias), selective reporting (reporting bias), and other biases. Each parameter was categorized as low risk of bias (+), high risk of bias (-), or unclear risk of bias (±).

### 2.5 Statistical Analysis

Statistical analyses were conducted using Stata software (version 15.0, StataCorp LLC, College Station, TX, USA). Between-study heterogeneity was quantitatively evaluated using Higgins’ I^2^ index, and a random-effects model was employed to pool effect sizes for placebo-controlled comparisons. Changes in blood pressure were expressed as standardized mean differences (SMD) percentages with 95% confidence intervals (CIs). Safety analyses were performed by calculating risk ratios (RRs) and 95% CIs. Between-study statistical heterogeneity was assessed based on I^2^ values. For all pooled results, a random-effects model was used when heterogeneity was high (I^2^ > 50%), and a fixed-effects model was used when heterogeneity was low (I^2^ < 50%). Begg’s test and Egger’s test were performed to identify small-study effects. For dichotomous data when performing Begg’s test and Egger’s test, a small constant (n = 0.5) is added to the zero-event group to enable effect size calculation. All tests were two-tailed, with *p* < 0.05 considered statistically significant.

## 3. Results

**Fig. 1.**
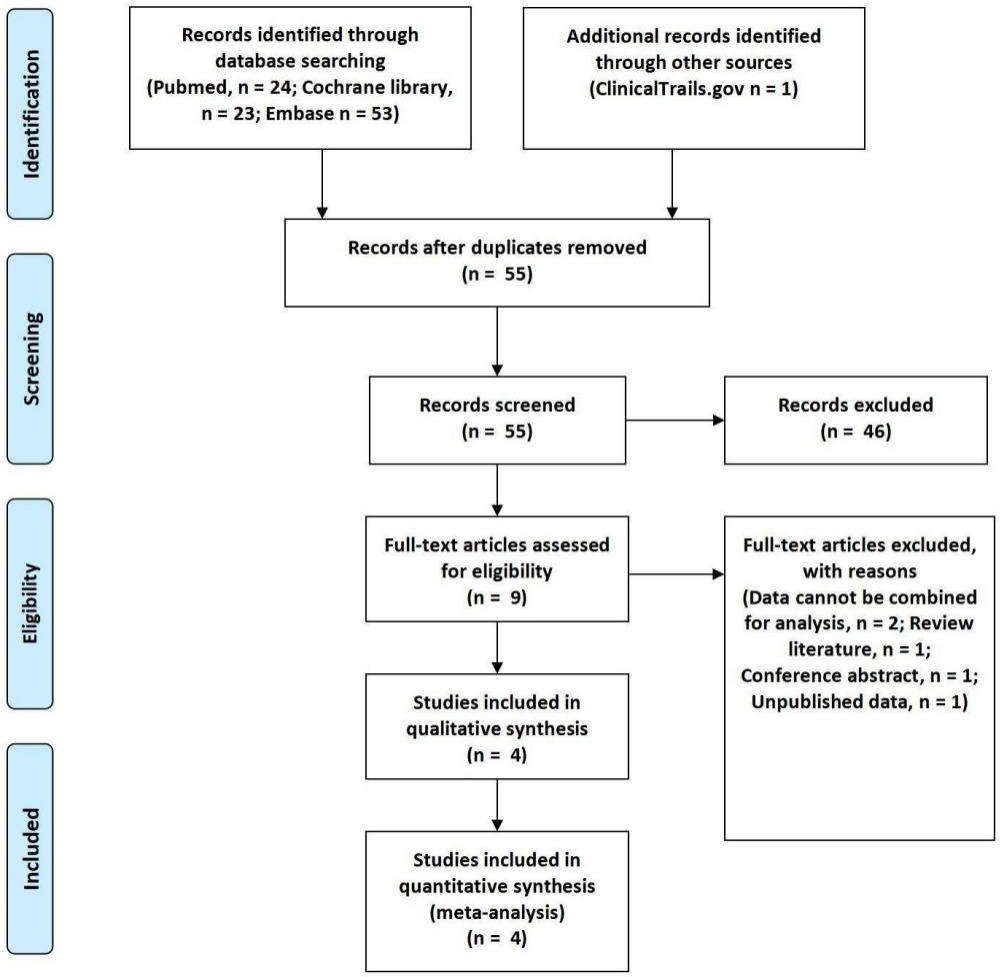
**Flow diagram**.

**Fig. 2.**
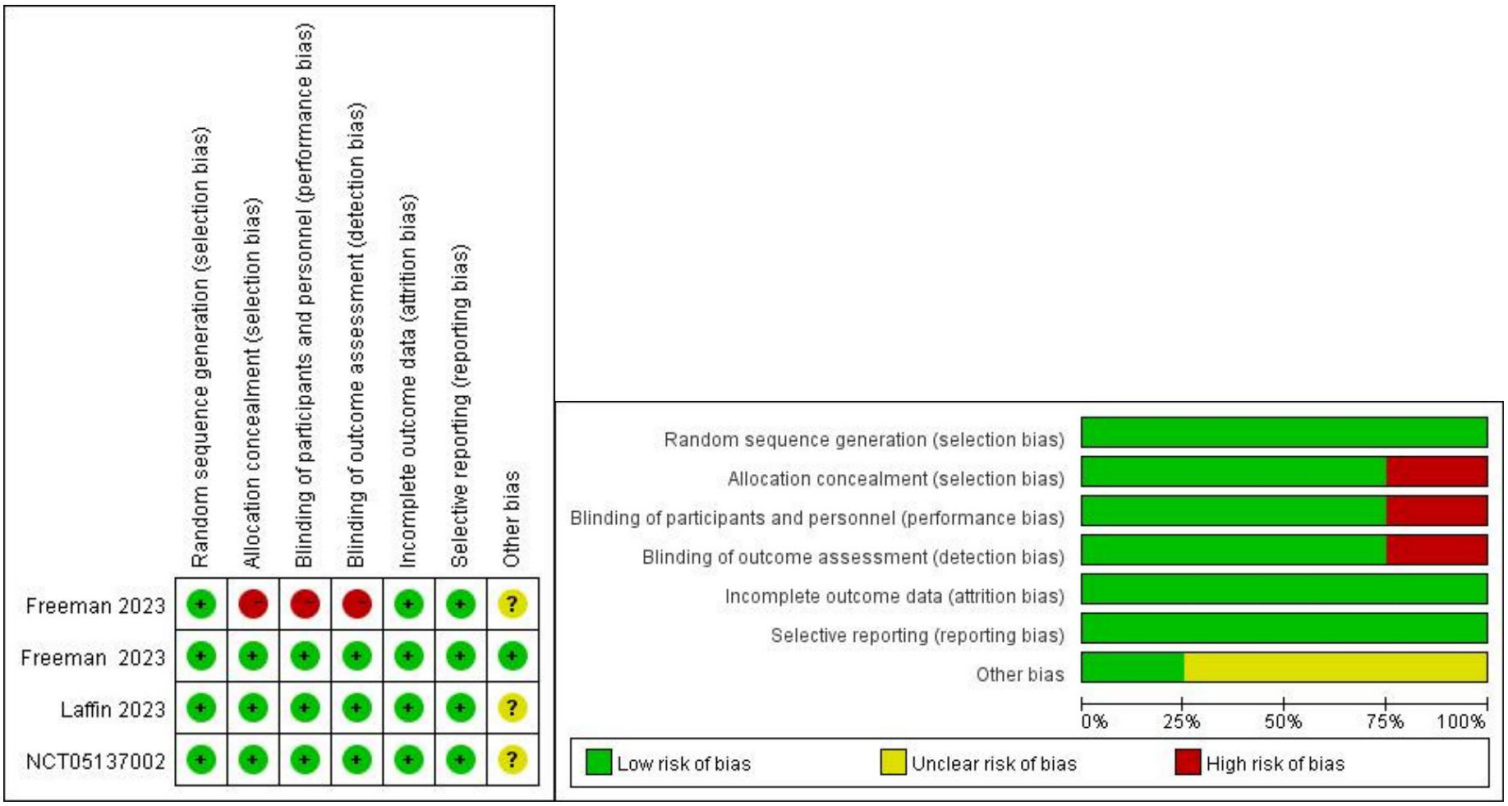
**Assessment of risk bias**.

This study ultimately included 4 trials comprising 776 participants [17, 18, 19, 20]. The detailed literature search process is illustrated in Fig. 1. The quality assessment results of the included studies are shown in Fig. 2. One study adopted an open-label approach with no concealment of allocation, posing a higher risk, while the remaining three studies employed blinding and concealment of allocation, with lower risk. The clinical trials used different doses of ASIs, with treatment durations ranging from 7 to 12 weeks. The selected study populations included individuals with RH and healthy adults. The characteristics of the included clinical trials are presented in Table 1.

**Table 1.** **Characteristics of the included studies**.

First author	Year	Country	Trial type	Phase	Duration (weeks)	Intervention	Control	Subjects	Financial support	Journal
Freeman	2023	USA	RCT	Phase 2	12	Baxdrostat	Placebo	Resistant Hypertension	CinCor Pharma	The New England Journal of Medicine
Freeman	2023	USA	RCT	Phase 1	7	Baxdrostat + Metformin	Metformin	Healthy Human	CinCor Pharma	American Journal of Cardiovascular Drugs
Laffin	2023	USA	RCT	Phase 2	8	Lorundrostat	Placebo	Resistant Hypertension	Mineralys Therapeutics	JAMA
NCT 05137002	2023	USA	RCT	Phase 2	8	Baxdrostat	Placebo	Resistant Hypertension	CinCor Pharma	Unpublished

Notes: RCT, Randomized controlled trial; NCT, National Clinical Trials.

We also extracted data for each subgroup, including baseline characteristics such as sex, age, SBP, DBP, and all reported adverse events, to observe the differences in baseline data between the intervention and control groups, thus assessing the differences in the baseline data in the population (Table 2).

**Table 2.** **Characteristics of the subjects at baseline**.

First author	Year	Case, n	Female, n (%)	Subgroup	Age (year)	SBP	DBP	Primary end points
Freeman	2023	275	153 (55.64)	Placebo	63.8 ± 10.8	148.9 ± 12.4	88.2 ± 6.1	SBP, DBP, AE
				Baxdrostat 0.5 mg	61.5 ± 10.3	147.6 ± 12.5	87.6 ± 7.7	
				Baxdrostat 1 mg	62.7 ± 10.1	147.7 ± 13.1	87.7 ± 6.0	
				Baxdrostat 2 mg	61.2 ± 10.8	147.3 ± 11.8	88.2 ± 7.1	
Freeman	2023	27	8 (29.63)	Baxdrostat 10 mg+M	-	-	-	AE
				M	-	-	-	
Laffin	2023	200	116 (58.00)	Placebo^&^	62.6 ± 10.7	142.7 ± 10.2	83.4 ± 8.8	SBP, DBP, AE
				Lorundrostat 100 mg OD^&^	68.7 ± 8.9	141.8 ± 10.9	78.9 ± 9.6	
				Lorundrostat 50 mg OD^&^	64.7 ± 9.5	140.0 ± 12.1	84.9 ± 6.4	
				Lorundrostat 25 mg TD^&^	64.8 ± 9.7	140.9 ± 9.6	78.9 ± 8.0	
				Lorundrostat 12.5 mg TD^&^	68.1 ± 10.1	144.6 ± 10.5	81.6 ± 7.6	
				Lorundrostat 12.5 mg OD^&^	65.2 ± 11.3	147.1 ± 11.6	82.4 ± 10.7	
				Placebo*	62.7 ± 12.3	140.4 ± 8.6	78.3 ± 7.6	
				Lorundrostat 100mg OD*	66.6 ± 10.6	132.9 ± 8.4	78.1 ± 7.6	
NCT 05137002	2023	249	132 (53.01)	Placebo	60.5 ± 10.6	147.9 ± 9.33	-	SBP, DBP, AE
				Baxdrostat 0.5 mg	59.9 ± 10.9	146.3 ± 8.60	-	
				Baxdrostat 1 mg	61.2 ± 10.7	147.0 ± 9.07	-	
				Baxdrostat 2 mg	59.2 ± 11.9	146.3 ± 7.83	-	

Notes: SBP, Systolic blood pressure; DBP, Diastolic blood pressure; AE, Adverse event; OD, Once daily; TD, Twice daily; M, Metformin. ^&^Plasma Renin Activity ≤1.0 ng/mL/h; * Plasma Renin Activity >1.0 ng/mL/h.

### 3.1 The Antihypertensive Effect of ASIs

**Fig. 3.**
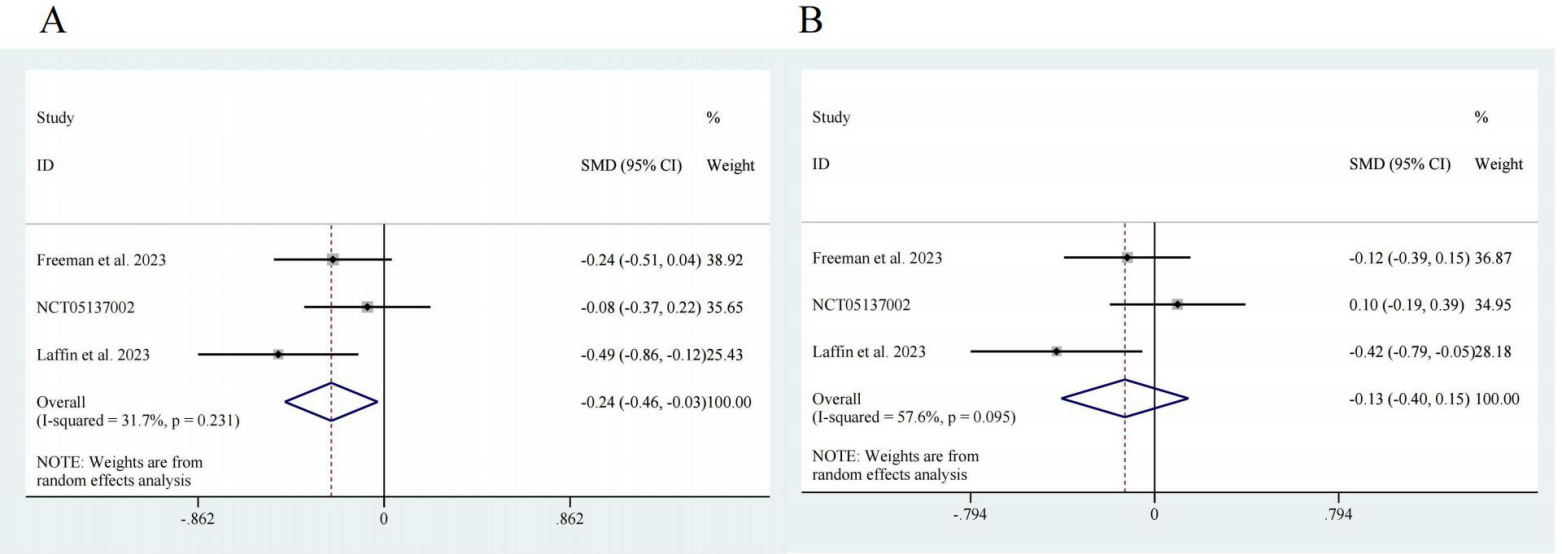
**Forest map**. (A) SBP, z = 2.23 *p* = 0.026. (B) DBP, z = 0.92 *p* = 0.359. CI, confidence interval; SMD, Standardized Mean Difference.

Three studies reported data on the efficacy of ASIs in RH patients, with 526 participants in the intervention group and 165 participants in the control group [17, 18, 20]. Regarding SBP, the pooled effect size was SMD = –0.24, with a 95% confidence interval (CI) of [–0.46, –0.03] and low heterogeneity (I^2^ = 31.7%). There was a significant difference (z = 2.23, *p* = 0.026) (Fig. 3A). For DBP, the pooled effect size was SMD = –0.13, with a 95% CI of [–0.40, 0.15] and high heterogeneity (I^2^ = 57.6%). There was no significant difference (z = 0.92, *p* = 0.359) (Fig. 3B).

### 3.2 Subgroup Analysis

**Fig. 4.**
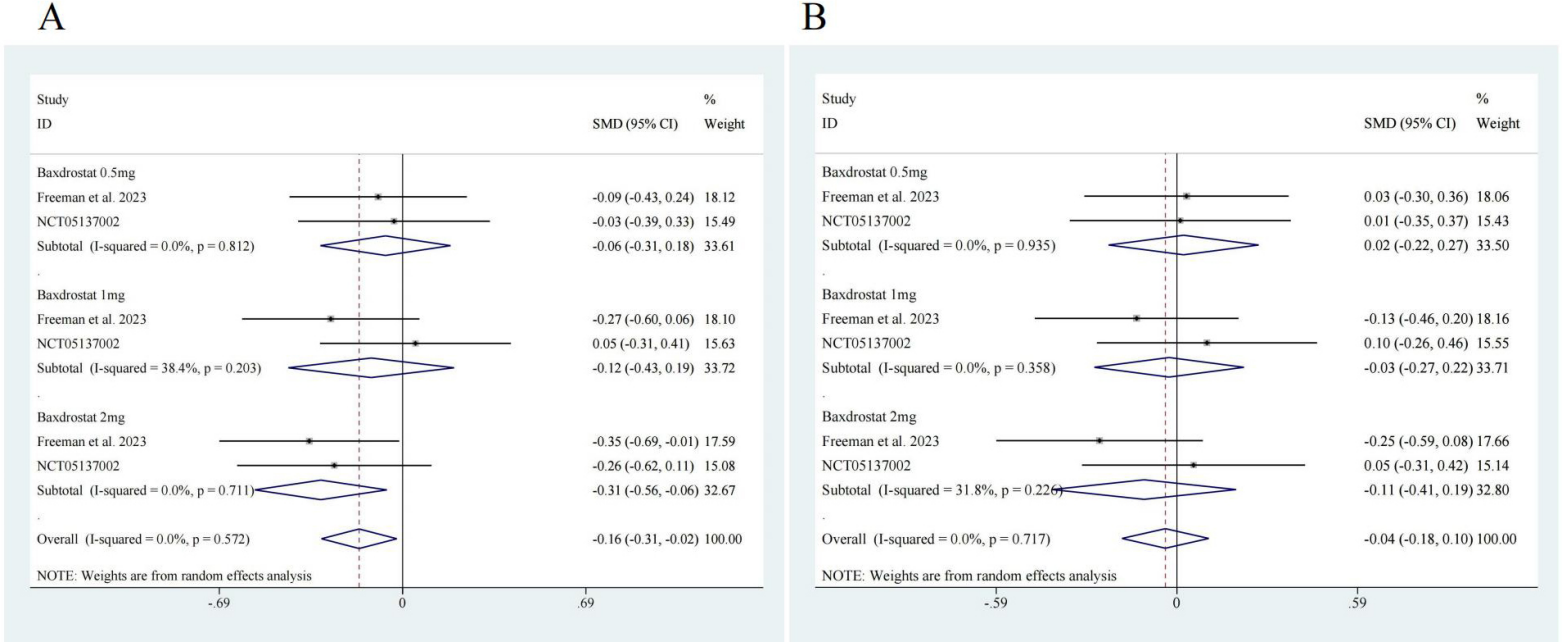
**Subgroup analysis forest map**. (A) SBP, ASI 0.5 mg, z = 0.52 *p* = 0.606; ASI 1 mg z = 0.74 *p* = 0.457; ASI 2 mg z = 2.43 *p* = 0.015; Overall z = 2.26 *p* = 0.024. (B) DBP, ASI 0.5 mg, z = 0.18 *p* = 0.861; ASI 1 mg z = 0.20 *p* = 0.838; ASI 2 mg z = 0.70 *p* = 0.485; Overall z = 0.52 *p* = 0.603. ASI, aldosterone synthase inhibitor.

To further evaluate the efficacy of ASIs, we performed subgroup analysis. Two studies reported data on different doses of ASI [17, 20], so subgroup analysis was conducted according to the dosage. The results showed that for SBP, 0.5 mg of ASI had an effect size of SMD = –0.06, with a 95% CI of [–0.31, 0.18], no significant heterogeneity (I^2^ = 0.0%) and no significant difference (z = 0.52, *p* = 0.606). A 1-mg dose of ASI had an effect size of SMD = –0.12, with a 95% CI of [–0.43, 0.19], with low heterogeneity (I^2^ = 38.4%) and no significant difference (z = 0.74, *p* = 0.457). A 2-mg dose of ASI had an effect size of SMD = –0.31, with a 95% CI of [–0.56, 0.06], no significant heterogeneity (I^2^ = 0.0%) and a significant difference (z = 2.43, *p* = 0.015). ASI had an overall effect size of SMD = –0.16, with a 95% CI of [–0.31, –0.02], no significant heterogeneity (I^2^ = 0.0%) and a significant difference (z = 2.26, *p* = 0.024) (Fig. 4A). For DBP, a 0.5-mg dose of ASI had an effect size of SMD = 0.02, with a 95% CI of [–0.22, 0.27], no significant heterogeneity (I^2^ = 0.0%) and no significant difference (z = 0.18, *p* = 0.861). A 1-mg dose of ASI had an effect size of SMD = –0.03, with a 95% CI of [–0.27, 0.22], no significant heterogeneity (I^2^ = 0.0%), and no significant difference (z = 0.20, *p* = 0.838). A 2-mg dose of ASI had an effect size of SMD = –0.11, with a 95% CI of [–0.41, 0.19], low heterogeneity (I^2^ = 31.8%), and no significant difference (z = 0.70, *p* = 0.485). The overall effect size of ASI was SMD = –0.04, with a 95% CI of [–0.18, 0.10], no significant heterogeneity (I^2^ = 0.0%), and no significant difference (z = 0.52, *p* = 0.603) (Fig. 4B).

### 3.3 Safety of ASIs

**Fig. 5.**
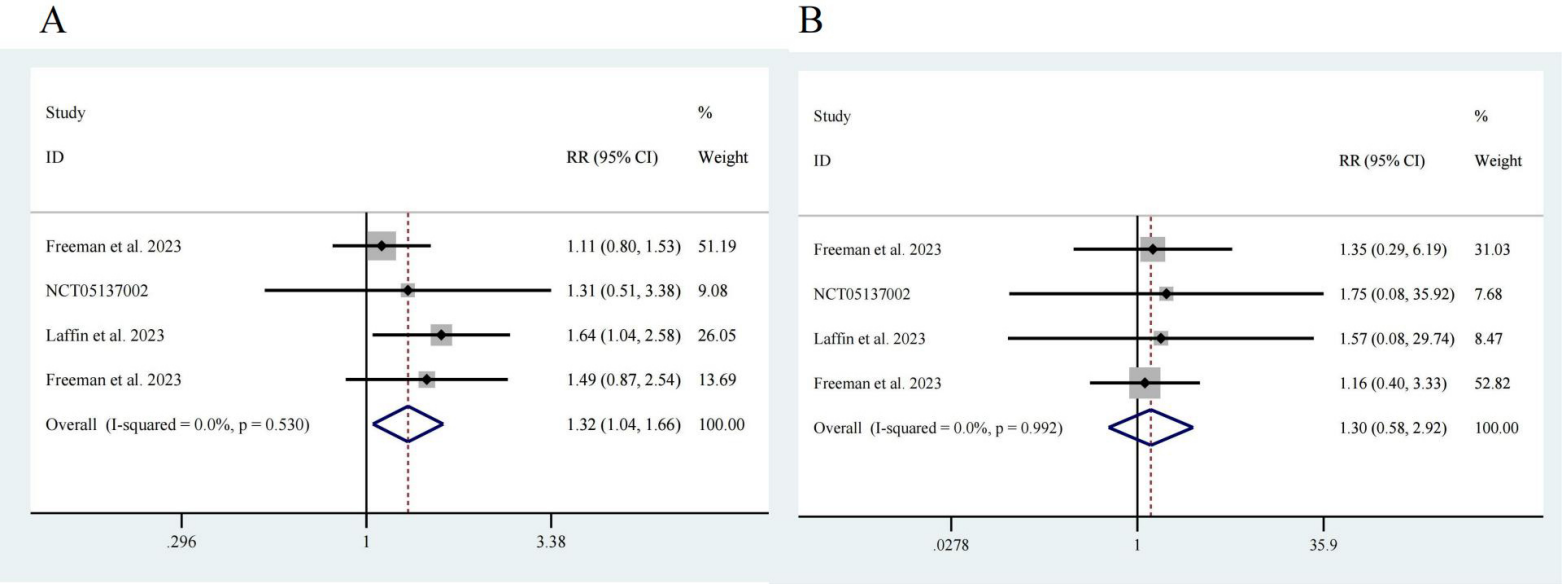
**Drug safety**. (A) Any adverse event, z = 2.32 *p* = 0.020. (B) Serious adverse event, z = 0.62 *p* = 0.532.

Next, we analyzed adverse events reported in the four studies [17, 18, 19, 20]. Among a total of 776 participants, 581 were in the intervention group, and 195 were in the control group. Notably, the risk of adverse events in the ASI group was greater than that in the control group, with an RR of 1.32 and a 95% CI of [1.04, 1.66], showing no significant heterogeneity (I^2^ = 0.0%) and a significant difference (z = 2.32, *p* = 0.02) (Fig. 5A). There was no significant difference in the incidence of serious adverse events between the participants receiving ASIs and those in the control group (z = 0.62, *p* = 0.532). The RR was 1.30, with a 95% CI of [0.58, 2.92], and there was no significant heterogeneity (I^2^ = 0.0%) (Fig. 5B).

### 3.4 Sensitivity Analyses

We performed sensitivity analyses by sequentially excluding each individual study during the evaluation of both primary outcomes and drug safety. The results demonstrated that the exclusion of any single study did not significantly alter the pooled estimates.

### 3.5 Publication bias

**Fig. 6.**
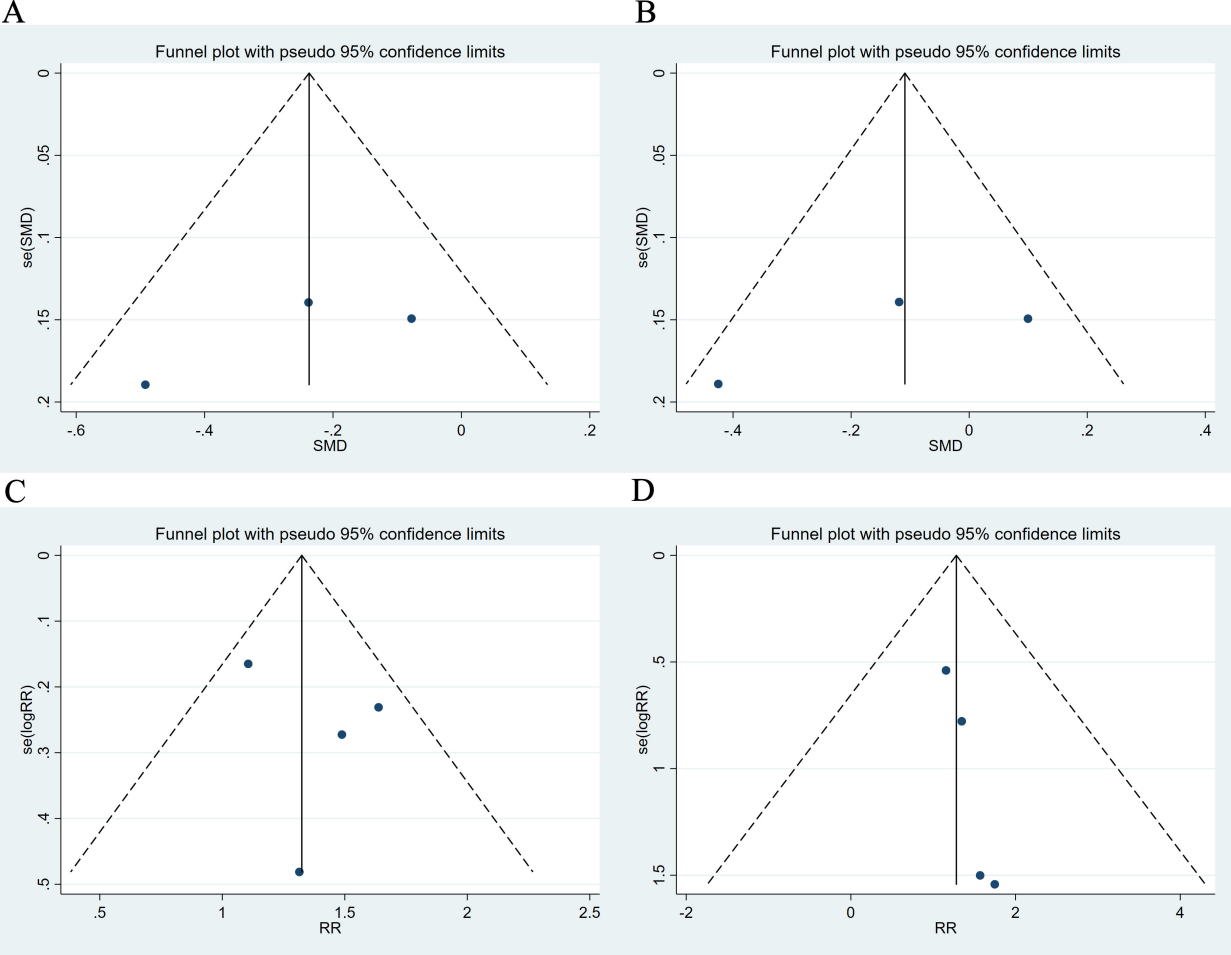
**Funnel plot**. (A) SBP. (B) DBP. (C) Any adverse event. (D) Serious adverse event.

We conducted an assessment of publication bias, and the results demonstrated a symmetrical funnel plot, indicating a low risk of bias in this study (Fig. 6). Begg’s test and Egger’s test did not indicate any small-study effects for SBP, DBP, any adverse event or serious adverse event (Table 3. All *p* > 0.05).

**Table 3.** **Small-study effects**.

	Begg’s test (*p* value)	Egger’s test (*p* value)
SBP	1.000	0.445
DBP	1.000	0.474
Any adverse event	0.734	0.203
Serious adverse event	0.308	0.425

## 4. Discussion

Patients with RH typically require full treatment compliance, which includes lifestyle modifications such as reducing sodium and alcohol intake, regular physical exercise, weight loss, and cessation of other factors that may interfere with blood pressure control [21, 22]. Aldosterone excess is common in patients with resistant hypertension, with primary aldosteronism observed in approximately 20% of diagnosed patients with resistant hypertension [23]. Excitingly, the emergence of ASIs in recent years has led to a reduction in the dose-dependent levels of 24-hour urine and serum aldosterone, an increase in plasma renin activity, and no decrease in serum cortisol, all of which support their selective mechanisms of action [24]. Although MRAs such as spironolactone and eplerenone have been validated in the treatment of resistant hypertension, their side effects (such as hyperkalemia and steroid-related adverse effects) limit their use in patients with chronic kidney disease. In contrast, aldosterone synthase inhibitors directly inhibit aldosterone synthesis, mitigating both genomic and non-genomic effects, and demonstrate improved safety and efficacy. For example, in the BrigHTN study, baxdrostat significantly reduced systolic blood pressure in patients with resistant hypertension, while presenting a lower risk of hyperkalemia [13]. Therefore, existing ASIs hold promise as novel options for lowering blood pressure in patients with refractory and uncontrolled hypertension [25].

The results of this study indicate that compared to placebo, ASIs can significantly improve SBP in patients with RH, with an SMD of –0.24 and a 95% CI of [–0.46, –0.03]. The efficacy of this class of new drugs is certain, and compared to other medications used for RH, ASIs also have more beneficial effects on urinary and serum aldosterone, renin, and cortisol levels [26, 27]. ASIs inhibit aldosterone production, thereby reducing the negative feedback on the renin-angiotensin-aldosterone system (RAAS) and leading to a compensatory increase in renin levels. This effect contributes to improved RAAS regulation and holds potential benefits in the treatment of RH. Specifically, the elevation of renin levels may enhance natriuresis, reduce sodium retention, and further support blood pressure control [26]. However, the results of this study show that aldosterone synthase inhibition has limited effectiveness in improving DBP (Fig. 3). Furthermore, our subsequent subgroup analysis also demonstrated similar differences in efficacy (Fig. 4). Generally, SBP and DBP represent cardiac output and peripheral vascular resistance, respectively. Aldosterone primarily affects sodium and water retention, which can lead to increased blood volume and, consequently, elevated systolic pressure. The effect on diastolic pressure, which is influenced more by vascular resistance, may be less pronounced due to the differing pathophysiological mechanisms involved [28]. SBP increases linearly with age, while DBP gradually increases and then declines around the age of 50, mainly due to changes in arterial stiffness and vascular compliance loss in older individuals, leading to increased SBP and decreased DBP [29]. It has been reported that higher plasma aldosterone concentrations are significantly associated with increased systolic blood pressure in subclinical hypercortisolism patients (adjusted difference [95% CI] = +0.59 [0.19–0.99], *p* = 0.008) but not in overt hypercortisolism patients [30]. Therefore, the differences in the efficacy of ASIs on SBP and DBP in RH patients may be related to plasma aldosterone concentrations.

Our study revealed that the risk of adverse events, primarily hyponatremia, hypotension, hyperkalemia, etc., was greater in the ASI group than in the control group [17, 18], while there was no significant difference in serious adverse reactions between the ASI group and the control group (Fig. 5). Mikhail’s [31] report indicates that various ASIs, including Baxdrostat, Lorundrostat, and BI690517, are associated with an increased risk of adverse events compared to placebo, such as hyperkalemia, hyponatremia, and renal impairment. Hyperkalemia and hyponatremia are primarily attributed to the reduction in aldosterone caused by ASIs, which decreases sodium reabsorption and potassium excretion in the epithelial cells of the distal tubules and collecting ducts. Renal impairment is hypothesized to be related to the diuretic effects of the drugs or a reduction in intraglomerular pressure. Overall, these drugs are relatively safe, with no reports of mortality or severe adverse events. Further validation of these findings awaits results from phase III trials [14]. Previous studies have indicated that RAAS inhibitors (RAASis) are commonly used drugs, and RAASis-related renal adverse events include hyperkalemia and acute kidney injury [32]. Moreover, aldosterone antagonists have been used as fourth-line antihypertensive drugs for the treatment of resistant hypertension in the past. The results also indicate that in patients with RH, compared to starting beta-blockers, the use of aldosterone antagonists significantly increases the risk of hyperkalemia, gynecomastia, and renal function deterioration [33]. In the study by Hanlon *et al*. [34], the incidence of serious adverse events in hypertensive patients taking RAAS antihypertensive drugs was much higher than that in the standard group (RR 3.70 [3.12–4.55]) and the elderly trial group (RR 4.35 [2.56–7.69]). A series of studies have evaluated the consequences of discontinuing RAASis (versus continuing) after an episode of hyperkalemia or acute kidney injury, consistently reporting worse clinical outcomes and higher risks of death and cardiovascular events [35]. Whether the latest ASIs pose similar risks remains unknown, and thus, further research is still needed this.

## 5. Limitations

Our study also has certain limitations. First, this study included only four trials with a limited sample size, which may introduce bias in the results. Therefore, it is important to avoid overinterpreting the existing data and to draw more conservative conclusions based solely on the current evidence. The included studies were not designed to assess the benefits and risks of aldosterone synthase inhibition beyond 12 weeks, nor were they intended to compare aldosterone synthase inhibition with other antihypertensive medications. Second, some studies included only patients with at least 70% compliance, potentially excluding patients with a lower response to antihypertensive medications. Additionally, due to limited data, this study did not specifically investigate the impact of patient background heterogeneity on the efficacy of aldosterone synthesis inhibitors. However, factors such as age, sex, comorbidities, genetic polymorphisms, and concomitant medications may influence treatment outcomes. Future research should focus on these factors to provide a more comprehensive understanding of the variability in treatment effects. Finally, the included studies did not explicitly state whether patients with primary aldosteronism (PA) were included. This issue may affect the generalizability and applicability of the results, especially since the efficacy of aldosterone synthase inhibitors in PA patients may differ from that in typical resistant hypertension patients. Future studies should clearly specify whether PA patients are included and explore their potential impact on treatment response to provide a more accurate assessment of therapeutic efficacy.

## 6. Conclusion

In summary, ASIs clearly have a certain efficacy in treating RH. These compounds exhibit antihypertensive effects on SBP, although their effects on DBP are limited. Notably, the risk of adverse events associated with ASIs was greater in the ASI group than in the control group, while there was no significant difference in severe adverse events between the ASI group and the control group. This may pose a certain obstacle that requires further research.

## Availability of Data and Materials

The data supporting the findings of this study are available from the corresponding author upon reasonable request.

## Supplementary Material

Supplementary material associated with this article can be found, in the online version, at https://doi.org/10.31083/RCM39555.
